# Decreased activity in zebrafish larvae exposed to glyphosate-based herbicides during development—potential mediation by glucocorticoid receptor

**DOI:** 10.3389/ftox.2024.1397477

**Published:** 2024-08-06

**Authors:** S. Spulber, L. Reis, P. Alexe, S. Ceccatelli

**Affiliations:** Department of Neuroscience, Karolinska Institutet, Stockholm, Sweden

**Keywords:** glyphosate, stress, glucocorticoid, developmental neurotoxicity (DNT), endocrine disruptors

## Abstract

Glyphosate-based herbicides (GBH) are a widely used group of pesticides that have glyphosate (GLY) as main active compound and are used to control a wide range of weeds. Experimental and epidemiological studies point to neurotoxicity and endocrine disruption as main toxic effects. The aim of this study was to investigate the effects of developmental exposure to GLY and GBH on locomotor behavior, and the possible contribution of GR-mediated signaling. We used zebrafish (*Danio rerio*) larvae in a continuous exposure regimen to GLY or GBH in the rearing medium. Alongside TL wildtype, we used a mutant line carrying a mutation in the GR which prevents the GR from binding to DNA (gr^s357^), as well as a transgenic strain expressing a variant of enhanced green fluorescent protein (d4eGFP) controlled by a promoter carrying multiple GR response elements (SR4G). We found that acute exposure to GBH, but not GLY, activates GR-mediated signaling. Using a continuous developmental exposure regime, we show that wildtype larvae exposed to GBH display decreased spontaneous activity and attenuated response to environmental stimuli, a pattern of alteration similar to the one observed in gr^s357^ mutant larvae. In addition, developmental exposure to GBH has virtually no effects on the behavior of gr^s357^ mutant larvae. Taken together, our data indicate that developmental exposure to GBH has more pronounced effects than GLY on behavior at 5 dpf, and that interference with GR-mediated signaling may have a relevant contribution.

## Introduction

Glyphosate-based herbicides (GBH) are a group of pesticides that have glyphosate (GLY) as main active compound and are used to control a wide range of weeds (Benbrook, 2016; Duke, 2018). GLY inhibits a plant-specific metabolic pathway, which produces secondary metabolites, and therefore has been claimed to be harmless to non-target organisms, such as humans and aquatic wildlife. However, several studies have linked GLY or GBH to a wide range of adverse health effects, including endocrine disruption (Pandey and Rudraiah, 2015; de Souza et al., 2017; Costa Reis et al., 2021; Milesi et al., 2021). The mechanisms of GLY and GBH toxicity are not thoroughly characterized, but experimental data points to endocrine disruption and neurotoxicity as main effects at low doses (Ames et al., 2022; Lopes et al., 2022). Neurotoxicity is of particular concern because of the association between occupational exposure to glyphosate and development of Parkinson’s Disease, as well as due to experimental studies documenting developmental neurotoxicity at doses below the limits set by regulatory agencies (Costas-Ferreira et al., 2022).

The glucocorticoid receptor (GR) controls the expression of several genes fundamental in endocrine signaling, participating in metabolism, inflammation, development, and stress response (Kadmiel and Cidlowski, 2013; Weikum et al., 2017). The effects of GLY and GBH on GR signaling had received limited attention to date. An interaction between GLY and stress hormone modulation of behavior has been suggested in tadpoles (Gabor et al., 2019), while in humans, GLY has been suggested to alter HPA axis function by selective effects on gut microbiota which promote cortisol secretion (Rueda-Ruzafa et al., 2019; Barnett et al., 2022). The aim of this study was to investigate the effects of developmental exposure to GLY and GBH on locomotor behavior, and the possible contribution of GR-mediated signaling. To this end we used zebrafish (*Danio rerio*) larvae in a continuous exposure regimen to GLY or GBH in the rearing medium. To investigate the possible effects on GR signaling, we used a mutant line carrying a mutation in the GR which prevents the GR from binding to DNA (gr^s357^, (Ziv et al., 2012; Facchinello et al., 2017)), as well as a transgenic strain expressing a variant of enhanced green fluorescent protein (d4eGFP) controlled by a promoter carrying multiple GR response elements (SR4G; (Krug et al., 2014)). We found that acute exposure to GBH, but not GLY, activates GR-mediated signaling. Using a continuous developmental exposure regime, we show that wildtype larvae exposed to GBH display decreased spontaneous activity and attenuated response to environmental stimuli, a pattern of alteration similar to the one observed in gr^s357^ mutant larvae. In addition, developmental exposure to GBH has virtually no effects on the behavior of gr^s357^ mutant larvae.

## Materials and methods

### Husbandry; mutant and transgenic strains

We used Tüpfel long-fin (TL) as wildtype control. To investigate the effect of GR-mediated signaling on the outcome of developmental exposure to GBH or GLY, we used the gr^s357^ strain, which carry a mutation in the glucocorticoid receptor preventing from binding to GRE sequences in the DNA (Ziv et al., 2012; Facchinello et al., 2017). The strain was imported from Max-Planck Institute of Neurobiology and maintained by homozygous crossings. To measure GR signaling, we utilized a transgenic strain that expresses enhanced green fluorescent protein (d4eGFP) under the control of a promoter region containing six of the most common GRE sequences found in the zebrafish genome (Tg (6xGRE:EGFP, myl7:TagBFP) mn48; SR4G) (Krug et al., 2014). The original strain was imported from Mayo Foundation for Medical Education and Research. For this study we have used a hybrid obtained by back-crossing SR4G males with TL females for at least 6 generations. All strains were maintained by the zebrafish core facility at Karolinska Institutet. In the holding facility, zebrafish were housed in self-cleaning 3.5 L tanks with a density of 5 fish per liter in a centralized recirculatory aquatic system (Tecniplast, Buguggiate, Italy). Basic water parameters were continuously surveilled and automatically adjusted to a temperature of 28°C; conductivity 1,200 μS/cm, pH 7.5. Other chemical water parameters were checked minimum monthly. The lightning scheme was 14 h light/10 h dark with a 20 min dawn and dusk period. Any animals are imported to a physically separate quarantine unit from which only surface-disinfected eggs are transferred to the breeding colony barrier. Health monitoring was done through Charles River according to the FELASA-AALAS guidelines (Mocho et al., 2022). *Mycobacterium chelonae* has been detected in sludge samples, ZfPV-1 in sentinel fish. Historically, *Pseudoloma neurophilia* had been detected in sentinels. Zebrafish embryos were staged according to Kimmel et al. (1995). All husbandry procedures are defined in SOPs which are available together with the latest health monitoring reports on request. Fertilized eggs were provided upon request. Breeding groups consisting of 3 males and 2 females were housed together overnight in 10 L spawning tanks with non-toxic plastic environmental enrichment. Fertilized eggs were collected 30 min after the light was turned on at 9:00 a.m. and stored at 28°C until further processing.

**FIGURE 1:**
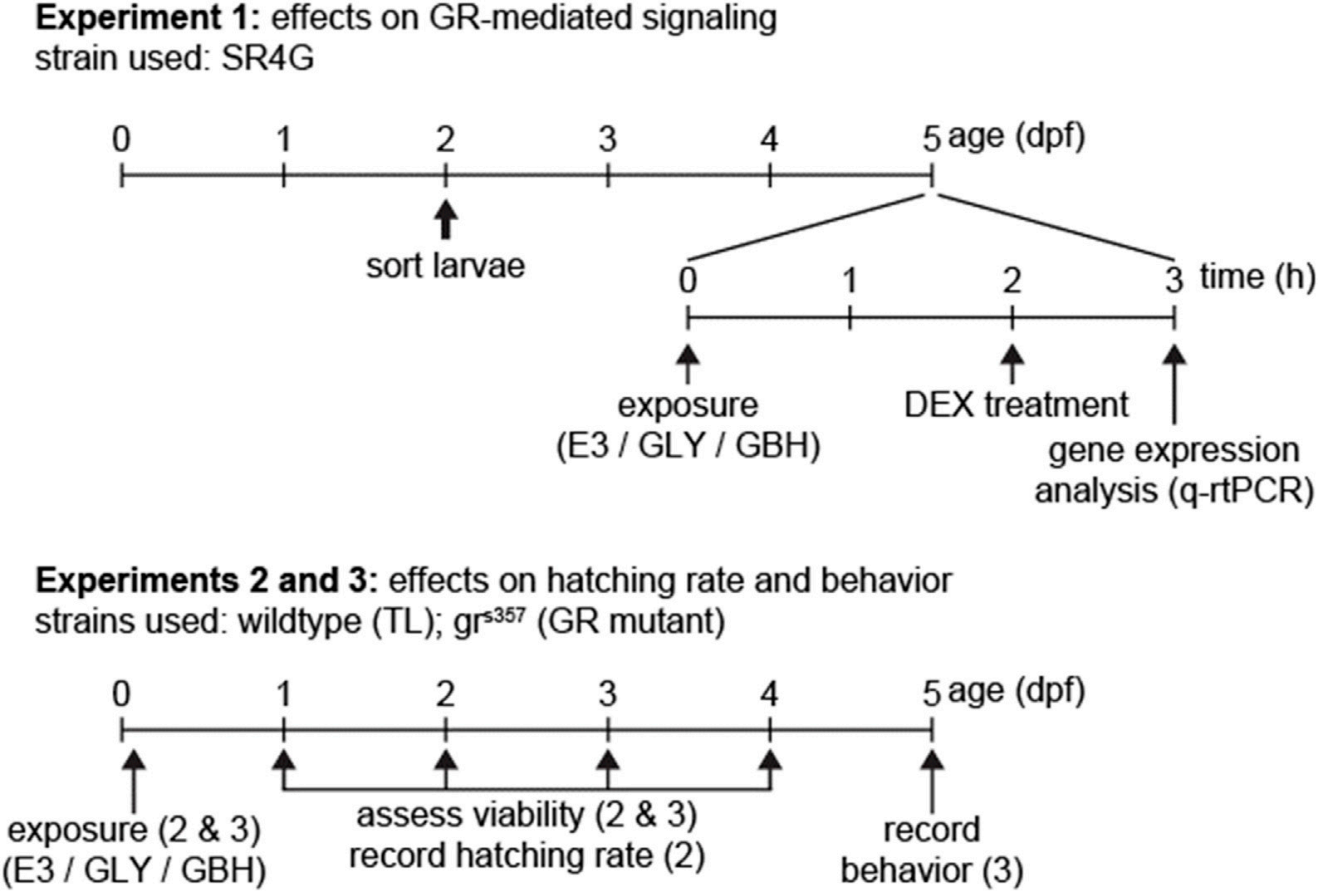
Timeline of experiments. In Experiment 1 we used exclusively SR4G (fluorescent reporter line for activation of GR-mediated signaling). For experiments 2 and 3, wildtype (TL) and GR mutants (gr^s357^) were used in parallel. Fertilization is depicted as timepoint 0 dpf.

A synoptic view of timeline and endpoints for experiments is provided in Figure 1. Experimental procedures pertaining to each experiments are described below.

### Gene expression analysis

To measure GR signaling, we utilized the SR4G reporter line (Krug et al., 2014). Fertilized eggs were obtained from 2 heterozygous SR4G females paired with 2 heterozygous SR4G males. The larvae were sorted before hatching at 2 dpf and only larvae with visible expression of blue fluorescent protein in the heart (Krug et al., 2014) were included in further experiments. The larvae were exposed to GBH or GLY at a concentration of 10 ppm for 3 h in the rearing water at the age of 5 dpf. We also used dexamethasone (DEX; a synthetic GR analog) as positive control for activation of GR-medicated signaling. After initiating the exposure, DEX treatment (final concentration: 10 mg/L in rearing medium) was added after 2 h to dishes with larvae exposed to 10 ppm GBH or GLY. The larvae were homogenized 3 h after initiating the exposure to GBH or GLY, respectively 1 h after DEX treatment. A total of ten larvae per group and biological replicate were used, and the experiment was repeated at least 5 times. In order to reduce stress after treatment, larvae were directly homogenized using BioMasher®-III homogenizers (Funakoshi Co. Ltd., Tokyo, Japan) and processed for RNA isolation using Trizol™ (Sigma-Aldrich). RNA was isolated from the homogenates using a Direct-zol™ MiniPrep kit (Zymo Research Corp, Irvine, CA, United States). The RNA was then reverse-transcribed into cDNA using a Maxima First Strand cDNA Synthesis Kit (ThermoFisher Scientific, Stockholm, Sweden) using 500 ng of template as follows: 10 min at 25°C, 25 min at 55°C, followed by 5 min at 85°C. The amplification steps were run using a QuantStudio™ 5 real-time PCR thermocyler (ThermoFisher Scientific). The relative gene expression of Glucocorticoid receptor (GR), d4eGFP, and GILZ was quantified by PCR real-time using beta-actin as housekeeping gene after confirming that its expression was not altered by the treatment (Supplementary Figure S1). Primer sequences: ActinB fw: CGA​GCA​GGA​GAT​GGG​AAC​C; rv: CAA​CGG​AAA​CGC​TCA​TTG​C (McCurley and Callard, 2008). GR, fw: ACC​ACT​TCA​AGC​GGA​CAG​AG, rv: CCG​GCT​TCT​GAT​CTT​TCT​GC (Dinarello et al., 2022). d4eGFP, fw: CGA​GCA​ACT​GAG​GAT​CCC​ATT​CTC​T, rv: CAC​CCC​GGT​GAA​CAG​CTC​CT (Krug et al., 2014). GILZ fw: CGA​CTT​GTT​TAT​ATG​GGC​TG; rv: TCT​TCA​GAC​ACC​AAC​ATG​CC.

### Developmental exposure

To remove all debris, the eggs were washed twice with fresh E3 medium (5 mM NaCl, 0.17 mM KCl, 0.33 mM CaCl2, 0.33 mM MgSO4; 0.05% methylene blue; pH 7.4) at room temperature on a clean lab bench under constant illumination. The zebrafish larvae were maintained in group of 20 larvae in 60 mm dish (VWR, Leuven, Belgium) with 5 mL E3 medium per dish at 28°C in a 10:14 h dark-light cycle (light on at 9 a.m.; 300 lx intensity, daylight-matching spectrum white light) until behavioral evaluation at 5 days post fertilization (dpf). Embryos were exposed to GBH (Roundup^®^ Transorb, Monsanto do Brasil LTDA, São Paulo Brazil, 480 g/L of glyphosate; other ingredients, reported as “inert compounds” by the manufacturer, are not known) or equivalent water solution of analytical standard glyphosate (480 g/L; GLY Sigma-Aldrich 45521) diluted in E3 medium. Control embryos were kept in E3 medium. The exposure to either GLY or GBH was initiated about 2 h post fertilization (hpf) (Figure 1). A static non-replacement regime exposure was applied according to OECD guidelines (*i.e.*, the rearing water was not replaced throughout the experiment). Using an inverted microscope (Nikon Ti-S) in brightfield configuration, the mortality, successful hatching, and embryonal malformations occurrence was assessed at the 24 (developmental failure/coagulation, light-induced coiling movements), 48 (developmental failure/coagulation, blood circulation, pericardial oedema, pigmentation), 72, and 120 hpf (hatching, eye development, swimming bladder inflation, morphological abnormalities such as scoliosis or bent spine) (see also Figure 1), as well as after completing the behavioral experiments. Larvae with morphological alterations (*e.g.*, bent spine, or deflated swimming bladder; ∼5% across all groups) at 5 dpf were excluded from analysis of behavior.

### Hatching

An equal number of fertilized eggs (n = 20) were placed in 6 cm Petri dishes in 5 mL E3 medium (∼1.7 mm depth), and hatching was evaluated by the same experimenter 2 h after turning the light on in the incubator every day between 1 and 4 dpf. Hatching time was evaluated in wildtype (TL) and gr^s357^ mutants by an assessor who was not blind to experimental conditions. The percentage of successful hatching for each timepoint was calculated after deducting the number of unfertilized eggs, and included the larvae with malformations documented after hatching (*e.g.*, bent spine or pericardial oedema).

### Activity monitoring and behavioral tests

The behavioral analysis on ZF larvae were conducted using a high throughput system, DanioVision™ (Noldus Information Technology, Wageningen, Netherlands), to record locomotor activity. The larvae were transferred to a 48-well plate (1 larva/well in 750 µL E3 medium; cylindrical wells, 10 mm inner diameter; VWR, Leuven, Belgium) on the day of behavior analysis. Each plate contained 8 larvae from all groups (wildtype and gr^s357^; control, 5 ppm and 10 ppm). The plates were transferred to the recording apparatus and maintained under constant environmental conditions for the larvae to accommodate for at least 1 h before starting the experiment. Inside the recording apparatus, the temperature was maintained at 28.5°C ± 0.2°C using a circulating water system, and white light (warm white spectrum; intensity at the bottom of the recording chamber: 3200–3500 lux) was provided throughout the experiment, except when testing the visual motor response (see below). Activity tracking was performed under infrared (IR) illumination using a camera (Basler ace acA1300-60 gm, BASLER AG, Germany) with IR filter on images were acquired at a rate of 50 frames per second (fps) (exposure time 5 ms). Automated image analysis was performed online using a dynamic subtraction algorithm with individual frame weight of 4%. Videotracking data were inspected for accuracy before analysis. The raw tracking data (xy coordinates), exported as ASCII files, were analyzed using custom routines in Matlab™ environment (The Mathworks Inc., Natick, MA, United States). To filter out detection noise, a bout of activity was defined as a continuous sequence of frames during which the displacement of the center of gravity was above the threshold (0.2 mm/frame). Bouts separated by less than 40 ms (2 frames) were considered as a single bout, and we calculated the frequency of occurrence, expressed in number of bouts per time unit (bouts/min), and the average distance moved within the bout (mm) as parameters for measuring the spontaneous activity.

The startle response is characterized by a brief surge in swimming activity (typically a single, more intense bout than spontaneous bouts) that occurs shortly after the application of a stimulus (vibrational, acoustic, electric, or tactile), followed by a period of inactivity before returning to spontaneous activity (Burgess and Granato, 2007a). The acoustic/vibrational startle response, in particular, develops in parallel with the auditory system, and consistent behavioral responses can only be observed in normal zebrafish larvae no earlier than 5 dpf (Zeddies and Fay, 2005; Tanimoto et al., 2009). To trigger the stimulus, we employed a customized system that includes a solenoid hitting the chassis of the recording chamber. The stimulus was activated from the video tracking software (EthoVision XT 10, Noldus) and synchronized with the behavioral observation. The larvae were allowed to habituate in the experimental setup for 4 min before applying the first stimulus. Each session included a series of ten equally spaced taps (1 min interval between taps). A startle response was validated only if the bout of activity was detected within 0.5 s (25 frames) after triggering the stimulus. The following parameters were calculated: average distance moved per second; latency to startle (measured as the interval between the stimulus application and the onset of the first subsequent bout); distance moved during the bout; and the period of inactivity after the startle response (measured as the delay between the end of the startle response and the start of the next bout) (Spulber et al., 2014). We did not observe any indication of habituation, such as a decreasing number of responsive larvae or a decreasing intensity of the startle response, in our system (data not shown). The probability of recording a startle response was calculated as the ratio between number of validated responses and the number of stimulations per session (the temporal order of responses was not considered). Group means were calculated by averaging the values for each parameter across the validated startle responses for each larva.

Freely moving zebrafish larvae exhibit the visual motor response (VMR), defined as a pronounced increase in swimming activity triggered by a sudden decrease in environmental light intensity (Burgess and Granato, 2007b; Emran et al., 2008). VMR can be assessed already in 4 dpf larvae, however, the spontaneous activity levels of the larvae vary considerably between individuals and cannot serve as a reliable baseline for measuring activity. Therefore, VMR was recorded when the larvae reached 5 dpf. Prior to the experiment, the larvae were acclimatized to the DanioVision™ chamber for at least 15 min, with the environmental temperature kept at 28°C using a custom-built temperature-controlled water system and the light intensity at 3200–3500 lux. Baseline activity was recorded during the last 10 min of the acclimatization period while the illumination remained constant. After acclimatization, the larvae were subjected to a 10-min dark pulse (no detectable light in the visible spectrum), with the transition from light to dark being nearly instantaneous. The larvae were killed with an overdose of tricaine (MS-222 > 900 mg/L) immediately after completing the behavior testing.

### Statistical analyses

Statistical analyses were performed in Statistica 14.0 (TIBCO Software Inc., Palo Alto, CA, United States). Activity data was analyzed using factorial ANOVA (main factors: dose, and experimental replicate; only effect of dose displayed) split by compound and genotype, followed by Dunnett’s *post hoc* test vs. control as reference group. Gene expression data was analyzed by one-sample *t*-test (significant gene expression regulation per group), and main effect ANOVA for exposure dose, split by compound, followed by Student’s t-test for planned paired comparisons. All results are shown as mean ± standard error of the mean.

## Results

The zebrafish larvae were exposed to increasing concentrations of GLY or GBH between 0.1 ppb and 10 ppm in E3 medium starting from 2 hpf, until the time of behavior testing at 5 dpf. The endpoints assessed were spontaneous activity, as well as the reaction to a dark pulse (visual motor response). We did not observe an increase in rate occurrence of malformations as compared to control larvae. The lowest concentration inducing significant changes in any parameter assessed was 5 ppm GBH, while consistent alterations across all parameters were detectable only at 10 ppm GBH (Supplementary Figure S2). For subsequent experiments, the zebrafish larvae were exposed to 5 ppm or 10 ppm.

### Acute exposure to GBH stimulates GR signaling

**FIGURE 2:**
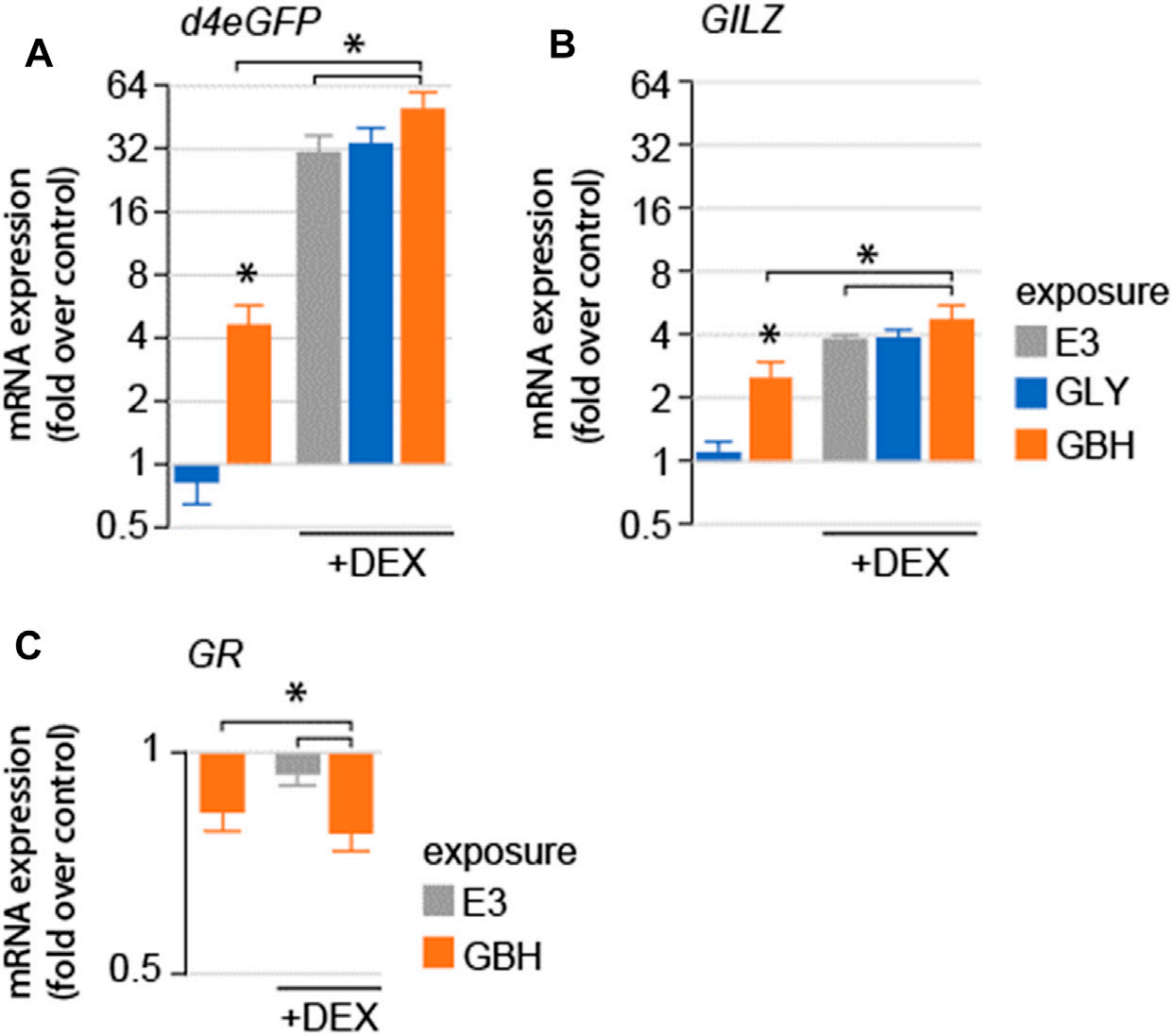
Effects of acute exposure to GLY or GBH on GR-mediated signaling in zebrafish larvae at 5 dpf. **(A)** Acute exposure to GBH, but to GLY upregulates the mRNA expression of GR-regulated genes (A–d4eGPF reporter system; **(B)** GILZ, endogenous GR-dependent gene). Exposure to GBH or GLY prior to DEX treatment does not modulate the effect or DEX treatment. The effects are consistent between reporter system expression (*d4eGFP*) and endogenous genes regulated by GR activation (*GILZ*). **(C)** mRNA expression for *GR* is downregulated following acute exposure to GBH, DEX treatment, as well as by DEX treatment following prior exposure to GBH.

We asked whether exposure to GLY or GBH alters GR-mediated signaling. To test this hypothesis, we used a mutant line expressing a reporter system consisting of *d4eGFP* expressed using a promoter containing 6 of the most common GR responsive elements identified in the zebrafish genome (SR4G) (Krug et al., 2014). As expected, DEX treatment dramatically upregulated the expression of *d4eGFP* mRNA (Krug et al., 2014) (Figure 2A). Acute exposure (3 h prior to homogenization) to GBH, but not GLY upregulated the expression of *d4eGFP* mRNA. Next, we used DEX as positive control for GR activation. The expression of *d4eGFP* mRNA was significantly upregulated by DEX treatment in all groups, however the upregulation was more robust in GBH-exposed larvae as compared to DEX treatment or GBH exposure alone (Figure 2A). To confirm the differential gene expression regulation observed in the reporter system, we also assessed the expression mRNA of glucocorticoid-induced leucine zipper protein (*GILZ*), a transcription factor expressed upon activation of GR (Wang et al., 2004) (Figure 2B). The expression of *GILZ* mRNA was significantly upregulated by DEX treatment and GBH exposure alone, but not by GLY exposure (Figure 2B). DEX treatment in GBH-exposed larvae resulted in significant upregulation of *GILZ* mRNA expression as compared to DEX treatment or GBH exposure alone (Figure 2B). One of the intracellular negative feedback mechanisms regulating the GR signaling is downregulation of *GR* mRNA expression. Therefore, we also evaluated GR mRNA expression and found significant downregulation in GBH-exposed larvae, as well as following DEX treatment (Figure 2C). In addition, DEX treatment of GBH-exposed larvae induced a more robust downregulation of GR mRNA as compared to either GBH-exposure or DEX treatment alone (Figure 2C).

### Exposure to GBH delays hatching

**FIGURE 3:**
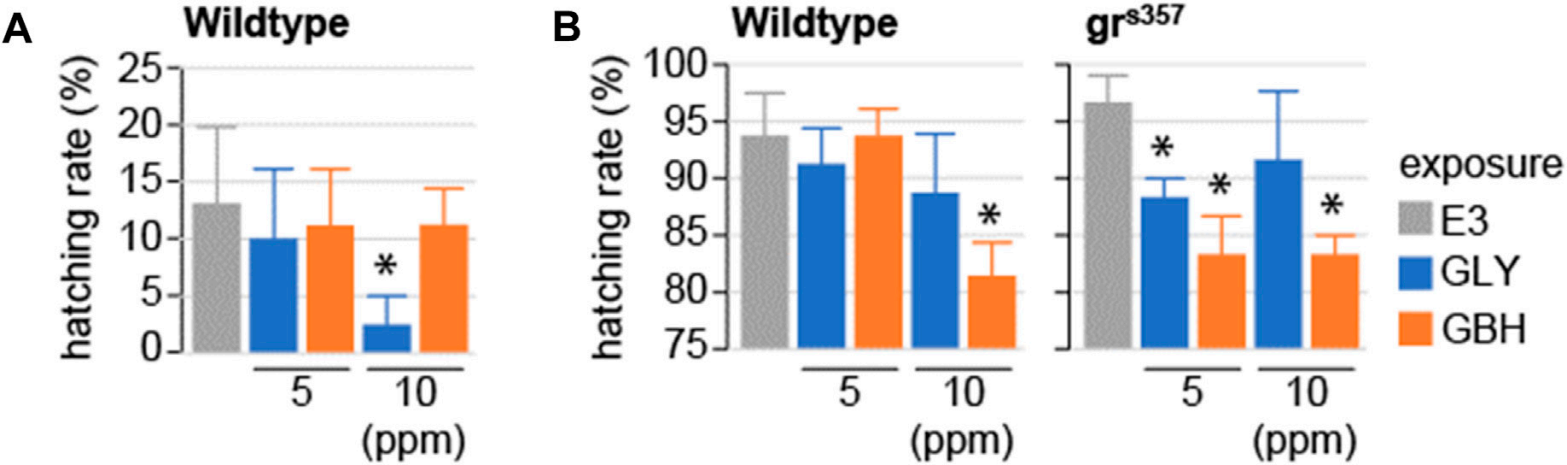
Effects of developmental exposure to GLY or GBH on spontaneous hatching. **(A)** Spontaneous hatching rate in 2 dpf wildtype larvae. No hatching is observed in gr^s357^ mutant larvae (not shown). **(B)** Spontaneous hatching rate at 3 dpf. Note the consistent decrease in hatching rate in mutant larvae exposed to either GLY or GBH. * - *p* < 0.05, Student’s t-test vs. Control (E3) (N = 3-4 experiments/group).

Hatching has been shown to be dependent on glucocorticoid signaling (Wilson et al., 2013). Of note, while gene expression regulation down-stream of GR signaling can be detected in zebrafish larvae before hatching, the GR activation is driven by maternal cortisol deposited before fertilization (Pikulkaew et al., 2011). We tested whether exposure to GLY or GBH during early stages of development impacts hatching. In wildtype larvae, hatching rate was significantly decreased at 2 and 3 dpf in larvae exposed to 10 ppm GBH, while exposure to GLY did not have any effect on hatching (Figure 3). To investigate whether the delayed hatching is mediated by interference with glucocorticoid signaling, we used homozygous larvae carrying a mutation in the glucocorticoid receptor which prevents it from binding to DNA–gr^s357^ (Ziv et al., 2012; Facchinello et al., 2017). Hatching is delayed in gr^s357^ larvae as compared to wildtypes, but the rate of hatching catches up already by 3 dpf (Figure 3). Exposure to either GLY (5 or 10 ppm) or GBH (10 ppm) in mutant larvae reduced hatching rate at 3 dpf (Figure 3). The delayed hatching was not associated with detectable morphological malformations in either wildtype or mutant larvae (not shown).

### Developmental exposure to GBH, but not GLY, suppresses spontaneous activity

**FIGURE 4:**
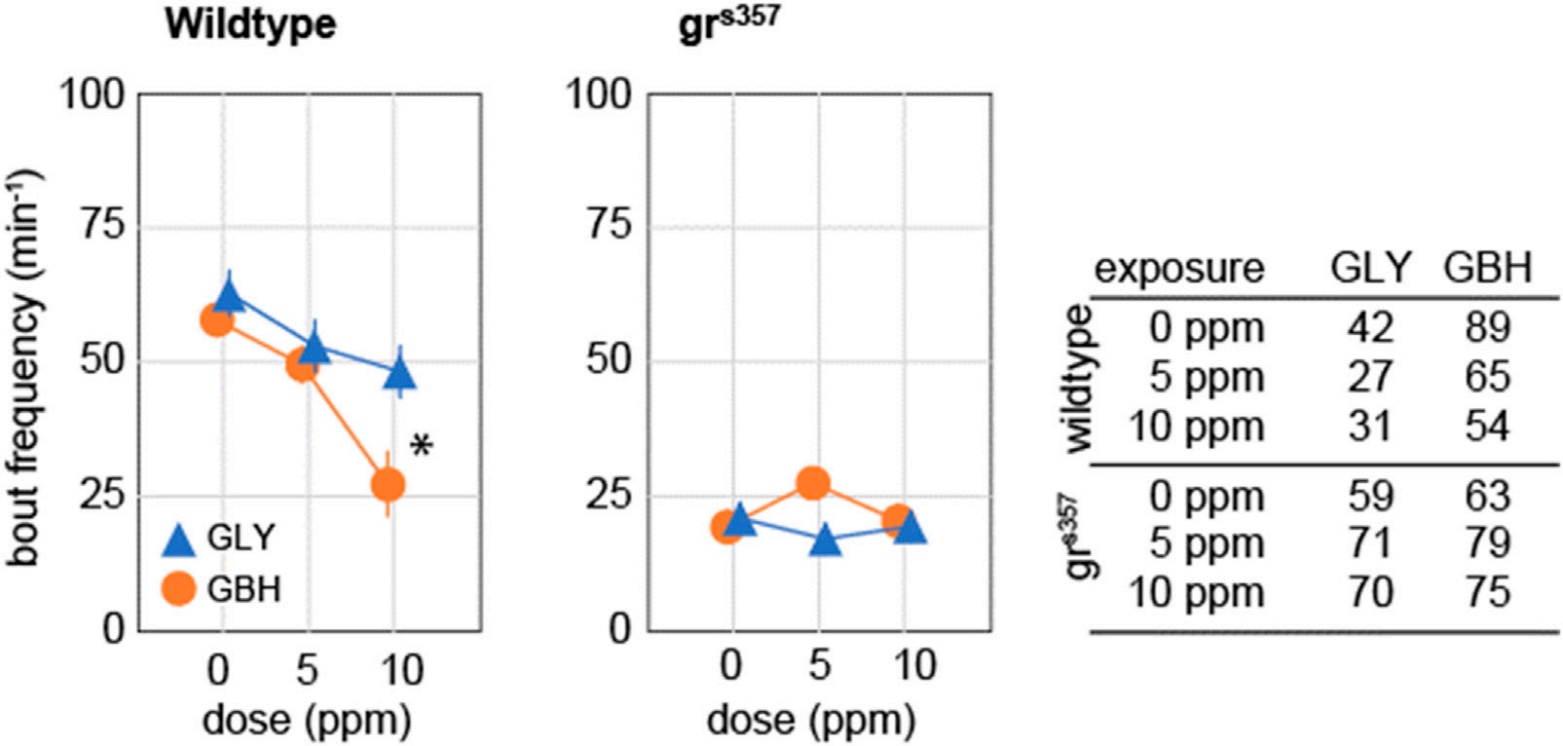
Effects of developmental exposure to GLY or GBH on spontaneous activity. Exposure to 10 ppm GBH reduces spontaneous activity in 5 dpf wildtype zebrafish larvae, but had no significant effect in gr^s357^ mutant larvae. GLY does not have significant effects on spontaneous activity in either wildtype of gr^s357^ mutant larvae. Note that gr^s357^ mutant larvae display greatly reduced frequency of spontaneous activity bouts as compared to wildtype larvae. The number of larvae recorded in each group is given in the table. * - *p* < 0.05, Dunnett’s *post hoc* test vs. controls (0 ppm).

Spontaneous activity in zebrafish larvae at 5 dpf consists of beat-and-glide sequences, with sparse, clearly identifiable bouts followed by intervals of complete immobility (Ingebretson and Masino, 2013; Spulber et al., 2014). We evaluated spontaneous activity as the frequency of swimming bouts averaged over 10 min of continuous tracking, and found that exposure to 10 ppm GBH, but not to GLY, significantly decreased the frequency of swimming bouts in wildtype larvae (Figure 4). Swimming bout frequency in gr^s357^ mutant larvae was lower than in wildtype larvae, and developmental exposure to either GBH or GLY did not have any significant effects (Figure 4).

### Developmental exposure to GBH, but not GLY, attenuates the response to environmental stimuli

**FIGURE 5:**
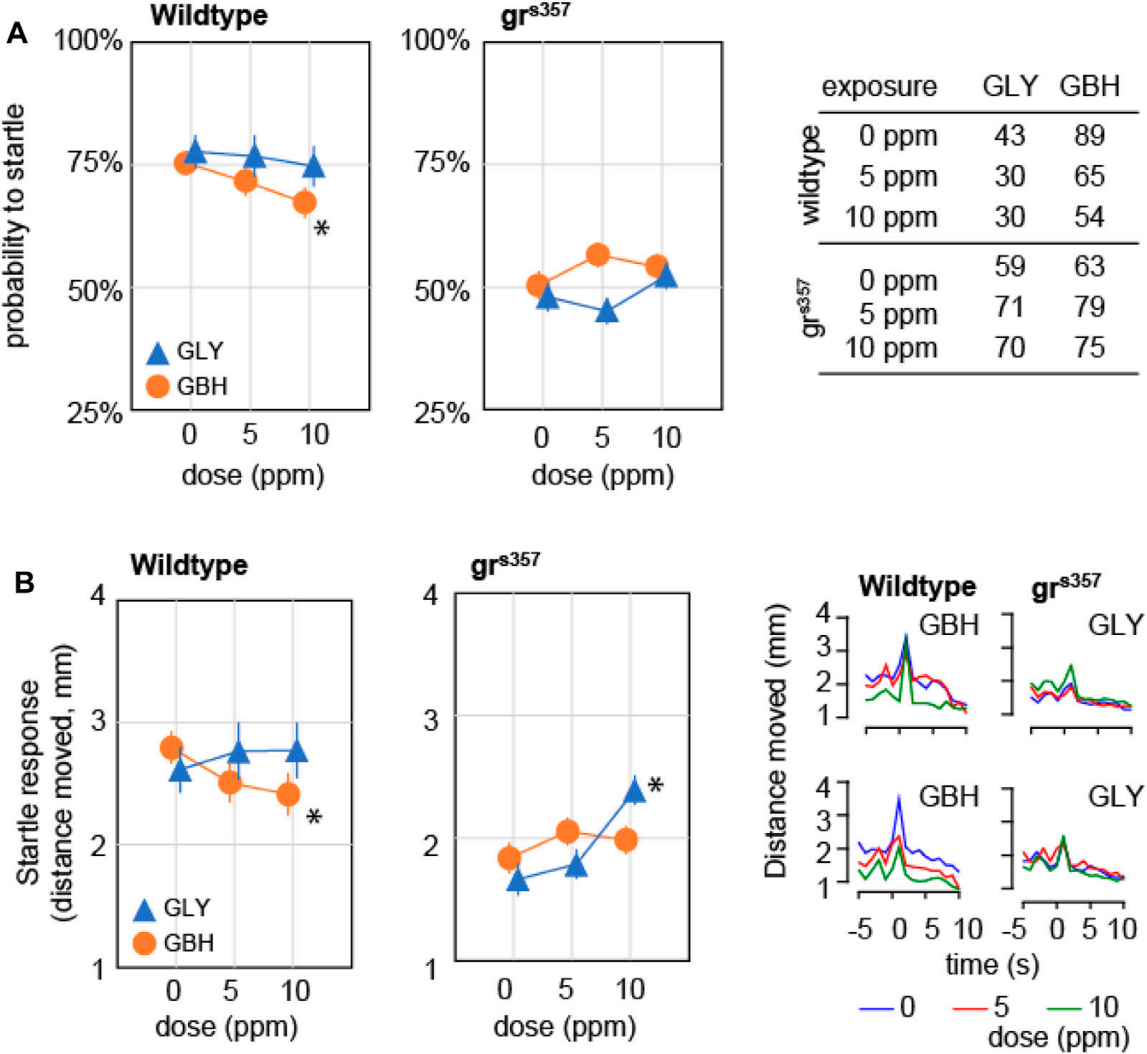
Effects of developmental exposure to GLY or GBH on acoustic startle response. **(A)** Effects on probability to mount a startle response for individual larvae. Exposure to 10 ppm GBH reduces the probability of recording a startle response in 5 dpf wildtype zebrafish larvae, while GLY did not have any significant effect in wildtype larvae. gr^s357^ mutant larvae have lower probability of recording a startle response, and neither GLY, nor GBH have significant effects. **(B)** Effects on amplitude of startle response. Wildtype larvae exposed to 10 ppm GBH display significantly lower amplitude of startle response than wildtype controls. In contrast, GBH exposure did not have significant effects in gr^s357^ mutant larvae, while larvae exposed to 10 ppm GLY display significantly stronger startle response as compared to mutant controls. The number of larvae recorded in each group is given in the table in panel **(A)**. * - *p* < 0.05, Dunnett’s *post hoc* test vs. controls (0 ppm).

We asked whether developmental exposure to GLY or GBH has any effect on behavioral responses to external stimuli. To this end we tested the startle response and the visual motor response (VMR). Startle response is defined as the reaction to a sudden stimulation of a sensory modality, and is visualized as a swimming bout considerably more intense than spontaneous bouts. The response to acoustic stimulation was evaluated as previously described (Spulber et al., 2014). The stimulation protocol was designed to prevent ceiling effect in wildtype larvae, therefore the individual probability to record a startle response is a meaningful measure. In wildtype larvae, the probability to startle was significantly decreased only in larvae exposed to 10 ppm GBH (Figure 5A), while exposure to GLY did not have a significant effect. The probability to record a startle response was significantly decreased in gr^s357^ mutant larvae as compared to wildtypes, while developmental exposure to either GBH or GLY had no significant effect (Figure 5A). The analysis of startle response amplitude revealed lower amplitude in wildtype larvae exposed to 10 ppm GBH, but higher amplitude in larvae exposed to 10 ppm GLY, while exposure to 5 ppm GBH or GLY did not have significant effects (Figure 5B). A detailed analysis of swimming bouts around the time of acoustic stimulation confirmed both the effects on spontaneous activity (decreased by GBH exposure), and on startle response (amplitude decreased by GBH exposure, and increased by GLY exposure). The amplitude of startle response was lower in control gr^s357^ mutant larvae than in control wildtypes, and the exposure to GBH did not have significant effects, while exposure to 10 ppm GLY significantly increased the startle response amplitude (Figure 5B). The analysis of swimming distance around the time of acoustic stimulation confirmed the overall decrease in spontaneous activity in gr^s357^ mutant larvae as compared to wildtypes, and the increase in startle response amplitude in gr^s357^ larvae exposed to 10 ppm GLY (Figure 5B).

**FIGURE 6:**
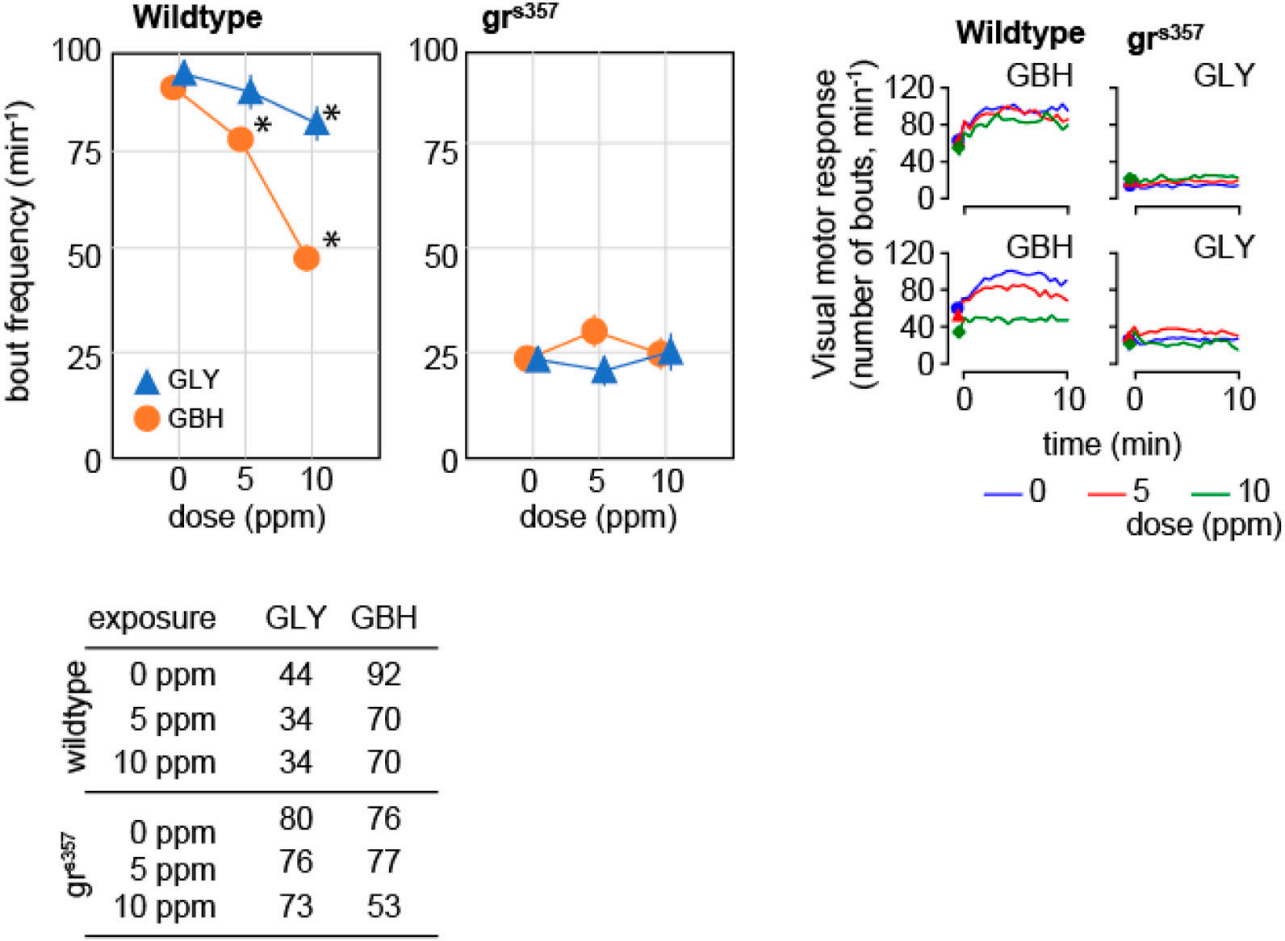
Effects of developmental exposure to GLY or GBH on visual motor response. Exposure to GBH suppresses the increase in swimming activity in response to a sudden change in environmental light intensity in a dose-dependent fashion in wildtype larvae. In contrast, only 10 ppm GLY exposure induces a significant decrease in visual motor response. Neither GBH, nor GLY have significant effects in gr^s357^ mutants. The number of larvae recorded in each group is given in the table in the bottom panel. * - *p* < 0.05, Dunnett’s *post hoc* test vs. controls (0 ppm).

VMR consists of a sustained increase in activity in response to a dark pulse administered during the light phase of light-dark cycle and has been assimilated to components of the stress response (Maximino and Herculano, 2010). The frequency of swimming bouts during the dark pulse increased by about 50% as compared to spontaneous activity in control wildtype larvae, and the increase was attenuated monotonically in a dose-dependent fashion in larvae exposed to GBH (Figure 6). In contrast, in wildtype larvae exposed to GLY, swimming bout frequency was decreased only in the 10 ppm (Figure 6). The analysis of distance moved during the dark pulse confirmed the dose-dependent effect of exposure to GBH, and highlighted the blunted increase in swimming bout frequency in wildtype larvae exposed to 10 ppm GBH (Figure 6). The visual motor response was greatly attenuated in gr^s357^ mutant larvae, and the difference from spontaneous activity was not significant (Figure 6). In addition, developmental exposure to either GBH or GLY did not have significant effects on visual motor response in gr^s357^ larvae (Figure 6).

## Discussion

The exposure dose we have used (up to 10 ppm) was in the range of concentrations measured in surface waters (Lopes et al., 2022), and did not induce anatomical or physiological abnormalities, in agreement with earlier reports (reviewed in (Ames et al., 2022)). We show that acute exposure to GBH, but not to pure GLY, activates GR-mediated signaling. Next, we assessed the effects of developmental exposure to GLY or GBH on swimming. We show that exposure to GBH leads to decreased spontaneous activity and attenuated response to stimuli, while exposure to pure GLY yields only sparse behavioral alterations. Using a GR-mutant strain, we show that the behavioral alterations induced by GBH in wildtype larvae are reminiscent of the effects of inactivation of GR signaling. In addition, we show that GBH exposure induces virtually no behavioral effects in GR-mutant larvae.

In this study we assessed the expression of genes regulated by GR activation following acute exposure to pure GLY or GBH in a reporter system transgenic zebrafish strain (SR4G) (Krug et al., 2014). Remarkably, we found significant upregulation in reporter gene expression only in GBH-exposed larvae, and the activation of gene expression downstream of GR activation was confirmed by the upregulation of endogenous genes (GILZ). The activation of GR-signaling by GBH exposure larvae is compatible with chronic inhibition of HPA/HPI (hypothalamic-pituitary-interrenal) axis function and can explain the observed alterations in swimming behavior.

The main effects of GBH on zebrafish larvae swimming have been summarized as reduced spontaneous activity and suppressed response to aversive stimuli (reviewed in (Lopes et al., 2022)). We have recorded swimming activity in three ethologically relevant conditions: spontaneous activity; response to acute sensory stimulation (startle response); and response to persistent changes in the environment (VMR). These conditions share the common feature of elementary “beat-and-glide” behavior, and the changes in swimming behavior in different conditions are due to modulation of amplitude and frequency of swimming bouts. The core difference between startle response and VMR resides in the central neuronal circuitry involved. The startle response is driven by a rather simple circuit centered on Mauthner cell activation (Burgess and Granato, 2007a). Cortisol has been shown to increase membrane excitability of Mauthner cells to facilitate escape responses under stress (Bronson and Preuss, 2017). In addition, we have shown recently that functional Mauthner cells are required for spontaneous swimming activity (Dehnisch Ellström et al., 2019). Therefore, the genetic ablation of GR signaling can explain the alterations we have observed in gr^s357^ mutants, namely, decreased spontaneous bout frequency, and decreased probability of recording a startle response. VMR is a more complex behavioral response which involves sustained activity in response to decrease in environmental light (Burgess and Granato, 2007b; Maximino and Herculano, 2010), and the behavioral adaptation requires the activation of stress response axis (Lee et al., 2018). In addition, constitutively reduced HPI activity has been shown to reduce the amplitude of VMR (Van Den Bos et al., 2017). An increase in cortisol secretion in response to external stressors, as well as changes in locomotor behavior in response to external stressors, can be consistently measured in zebrafish larvae starting at 5 dpf (Lee et al., 2018). We found a dramatically attenuated VMR in gr^s357^ mutants as compared to wildtype larvae. In wildtype larvae exposed to GBH, the alterations in swimming behavior resemble the alterations observed in gr^s357^ mutants: delayed hatching, decreased spontaneous activity, and attenuated response to external stimuli. In addition, GBH-exposed gr^s357^ mutant larvae display virtually no behavioral alterations, suggesting that GR-mediated signaling is required for the behavioral alterations to be measurable. The phenotype is much less attenuated in GLY-exposed larvae: we could only document a small decrease in VMR in larvae exposed to 10 ppm GLY. This can be explained by the co-formulants’ contribution to increase the absorption of glyphosate in GBH. Alternatively, unspecified co-formulants may directly induce stress in zebrafish larvae (Mesnage et al., 2022; Makame et al., 2023).

In humans, glyphosate has been suggested to alter HPA axis function by selective effects on gut microbiota which promote cortisol secretion (Rueda-Ruzafa et al., 2019; Barnett et al., 2022). However, while glyphosate is the cognate measurable analyte, occupational exposure is due to usage of commercial formulations. Therefore, one cannot exclude relevant contributions from co-formulants (Gandhi et al., 2021; Martins-Gomes et al., 2022). Here we show that behavioral alterations are pervasive following exposure to GBH, while exposure to GLY yields only sparse effects. Taken together, our data suggest a potential direct interaction between GLY and GR signaling, as has been shown for a wide range of environmental pollutants, including methylmercury (Ahir et al., 2013; Spulber et al., 2018). The conspicuous differences between effects of exposure to GLY or GBH point to an amplification of effects of the active ingredient in the commercial formulation. In conclusion, our data indicate that an investigation of potential disruption of HPA axis is a meaningful addition to epidemiological studies investigating the neurotoxic effects of occupational exposure to glyphosate.

## Data Availability

The data presented in the study are deposited in the BioStudies repository, accession number S-BSST1464 (https://www.ebi.ac.uk/biostudies/studies/S-BSST1464).
