# Supplementary material for: Decreased activity in zebrafish larvae exposed to glyphosate-based herbicides during development—potential mediation by glucocorticoid receptor
Source: Front Toxicol. 2024 Aug 6;6:1397477. doi: 10.3389/ftox.2024.1397477 (PMC11333450; doi:10.3389/ftox.2024.1397477)

Decreased activity in zebrafish larvae exposed to glyphosate-based herbicides during development – potential mediation by glucocorticoid receptor

Spulber S, Reis L, Alexe P, Ceccatelli S

Department of Neuroscience, Karolinska Institutet, Stockholm, Sweden

Supplementary Figure S2. Dose-range finding. Zebrafish larvae were continuously exposed to increasing doses of GLY or GBH starting at 2 h post fertilization (2 hpf) until the time of testing (5 dpf). We monitored the effects on spontaneous and stimulated (visual motor response) activity to capture the dose range where significant effects are detectable. Note that spontaneous activity is reduced following exposure to 10 ppm GBH, while sustain stimulated activity is reduced by both 5 and 10 ppm GBH, and only by 10 ppm GLY. The rate of occurrence of malformations was not increased in larvae exposed to GLY or GBH during development. Therefore, we used the lowest doses yielding significant effects on behavior for further investigations.

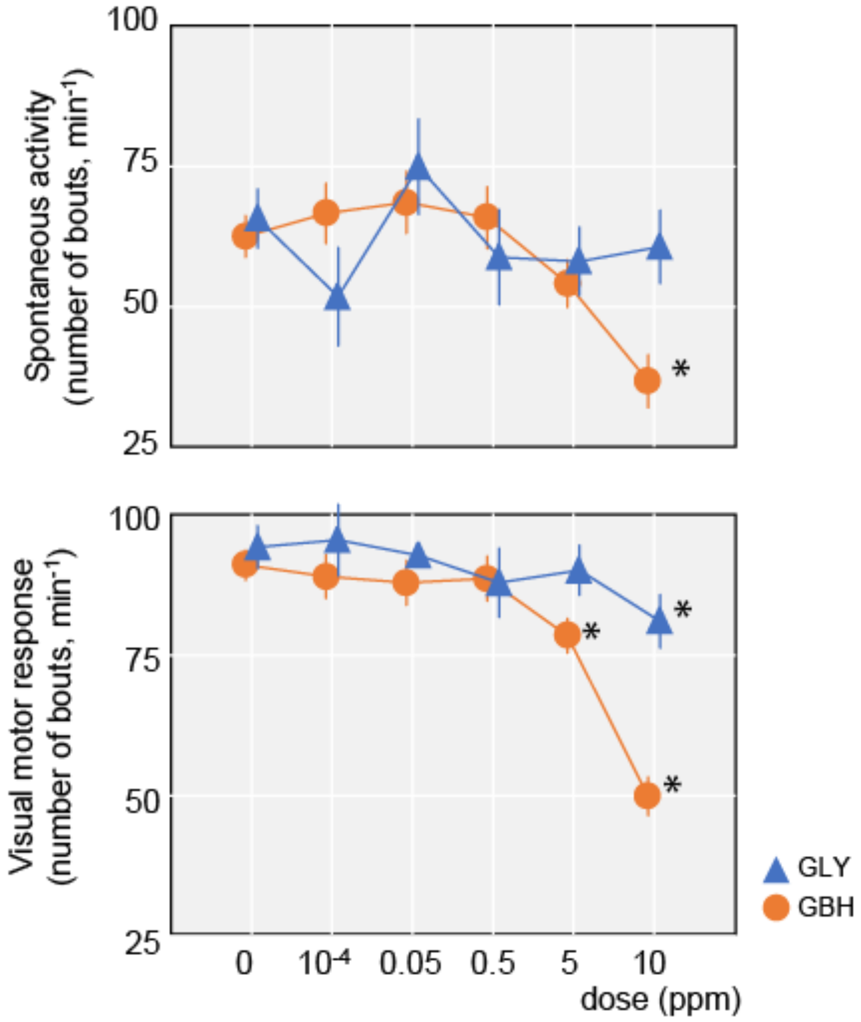

Supplement: Supplementary file 1 [file Image2.pdf]
