# Supplementary material for: Decreased activity in zebrafish larvae exposed to glyphosate-based herbicides during development—potential mediation by glucocorticoid receptor
Source: Front Toxicol. 2024 Aug 6;6:1397477. doi: 10.3389/ftox.2024.1397477 (PMC11333450; doi:10.3389/ftox.2024.1397477)

Decreased activity in zebrafish larvae exposed to glyphosate-based herbicides during development – potential mediation by glucocorticoid receptor

Spulber S, Reis L, Alexe P, Ceccatelli S

Department of Neuroscience, Karolinska Institutet, Stockholm, Sweden

Supplementary Figure S1. Expression of housekeeping gene (beta actin). No significant difference in absolute levels of expression as estimated by the cycle number when reaching the quantification threshold.

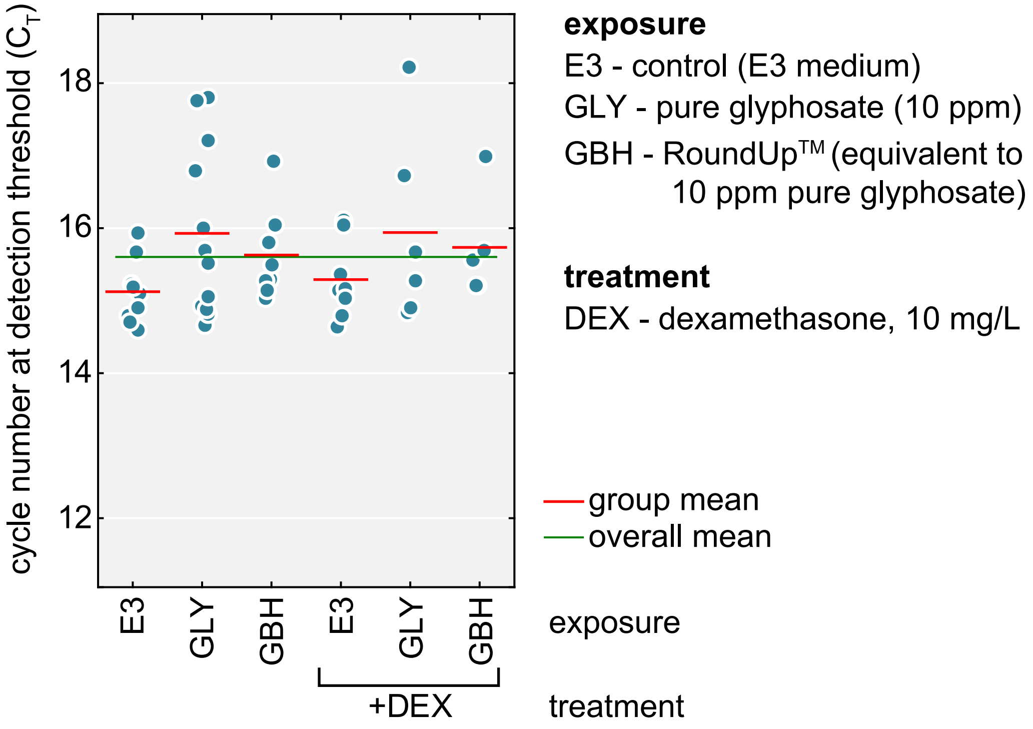

Supplement: Supplementary file 2 [file Image1.pdf]
